# Mindfulness-Based Interventions for Physical Conditions: A Narrative Review Evaluating Levels of Evidence

**DOI:** 10.5402/2012/651583

**Published:** 2012-11-14

**Authors:** Linda E. Carlson

**Affiliations:** ^1^Division of Psychosocial Oncology, Department of Oncology, Faculty of Medicine, University of Calgary, Calgary, AB, Canada T2N 4N2; ^2^Department of Psychosocial Resources, Tom Baker Cancer Centre, Alberta Health Services Cancer Care, Calgary, AB, Canada T2S 3C1

## Abstract

Research on mindfulness-based interventions (MBIs) for treating symptoms of a wide range of medical conditions has proliferated in recent decades. Mindfulness is the cultivation of nonjudgmental awareness in the present moment. It is both a practice and a way of being in the world. Mindfulness is purposefully cultivated in a range of structured interventions, the most popular of which is mindfulness-based stress reduction (MBSR), followed by mindfulness-based cognitive therapy (MBCT). This paper begins with a discussion of the phenomenological experience of coping with a chronic and potentially life-threatening illness, followed by a theoretical discussion of the application of mindfulness in these situations. The literature evaluating MBIs within medical conditions is then comprehensively reviewed, applying a levels of evidence rating framework within each major condition. The bulk of the research looked at diagnoses of cancer, pain conditions (chronic pain, low back pain, fibromyalgia, and rheumatoid arthritis), cardiovascular disease, diabetes, human immunodeficiency virus (HIV)/acquired immune deficiency syndrome (AIDS), and irritable bowel syndrome. Most outcomes assessed are psychological in nature and show substantial benefit, although some physical and disease-related parameters have also been evaluated. The field would benefit from more adequately powered randomized controlled trials utilizing active comparison groups and assessing the moderating role of patient characteristics and program “dose” in determining outcomes.

## 1. Introduction, Scope, and Chapter Outline

This paper summarizes the growing literature investigating the application of mindfulness-based interventions (MBIs) for people coping with a wide array of physical diseases and conditions. Mindfulness can be defined as the application of nonjudgmental present-focused awareness to the totality of experience moment by moment [1, 2]. This is often contrasted to the typical state of mental activity, which is to be thinking about the past, planning for the future, or analyzing and processing current experience, often with a tone of judgment or impatience. Mindfulness is both a practice, but also a way of being in the world. In formal mindfulness mediation practice, the focus is often purposefully directed towards the breath, body sensations, feelings, or thoughts, but it can also be simply bare awareness of whatever arises into consciousness moment by moment. Mindfulness practices emphasize not only the aspect of focusing awareness in the present moment, but also the attitudes with which attention is applied, encompassing acceptance, patience, openness, curiosity, kindness, and nonstriving [1].

Although originating from a 2,500 year old Buddhist tradition, modern meditation practices are often cultivated in a secular medical environment through structured training programs known as MBIs. MBIs in this area include mindfulness-based stress reduction (MBSR) [1], mindfulness-based cognitive therapy (MBCT) [3], acceptance and commitment therapy (ACT) [4] and other modifications or variations on these that incorporate mindfulness training. While dialectal behavior therapy (DBT) also includes some training in mindfulness practices, training in MBIs is not the primary focus, and most research has been conducted in mental health populations, rather than in people primarily suffering from a medical condition. For this reason DBT studies were not included in this paper.

MBSR, the most commonly applied intervention, is typically an 8-week group program which focuses on training in a variety of mindfulness meditation practices including sitting meditation, body scan, walking meditation, and loving kindness meditation. It also incorporates a large component of gentle Hatha yoga, practiced as “mindful movement.” Dialogue and inquiry into the process of learning mindfulness is also a key element of the intervention which promotes self-discovery and personal growth. For purposes of this paper, studies of yoga interventions alone, while arguably incorporating elements of mindfulness, were excluded, due to the volume of work accumulating in each area. Studies focusing largely on process variables were also excluded, as the focus was on the clinical efficacy of MBIs for treating both psychological and physical symptoms related to the conditions under study.

This paper could easily become simply a catalogue or list of conditions, studies, and outcomes, detailing what changes and to what extent after people participate in mindfulness-based interventions. A catalogue of research results such as this summarizes the current state of the science, but quickly becomes outdated in the prevailing fast-paced research environment. To satisfy those readers who are seeking such a summary, and to provide a comprehensive reference document, the bulk of this work will be summarized here, and the quality of the evidence rated. However, the approach at the outset is to consider more deeply why mindfulness-based interventions might be helpful to people suffering from a wide array of physical symptoms, diseases, and conditions.

The questions that are important to address at the outset are the following. What do all these conditions have in common? Could there be a possible underlying reason or mechanism by which the application of mindfulness may be helpful across such a diverse array of problems? There may not be one unifying underlying concept, but several possibilities merit exploration. Hence, the first part of this paper will discuss issues common across many medical conditions that MBIs may address, which will be followed by a summary of the research to date in this broad area. Synthesis of the research and analysis of the state of the science and quality of research methodology in general will be offered, focusing on evaluating the level of evidence across interventions, concluding with recommendations for further research in this area.

## 2. What Happens When Diagnosed with a Physical Health Condition?

 To understand the potential benefits of mindfulness in physical medical conditions it may be helpful to think about the illness experience itself. For example, consider the case of cancer, which many would agree may represent the most extreme jolt to a person's view of themselves and the world they live in, not to mention having to deal with the practical implications of having to take time away from career and family for debilitating and often mutilating treatments. On an existential level people are forced to confront their own mortality in a way many have never considered before. Whether the diagnosis is cancer, heart disease, or chronic pain, the possibility of one's death becomes real and potentially imminent, and substantial and perhaps permanent changes in functional abilities and lifestyle may follow.

While we are all intellectually aware that we may die at any time, that awareness is typically not acute; it is not felt daily in the body as an imminent threat. We do not really live our lives believing we may perish at any moment, which is generally adaptive. For people diagnosed with acute or even chronic illness, many are forced to encounter this reality directly for the first time. It can cause fear, terror in fact, and challenges ones beliefs about control and certainty. Most people walk through life mistakenly believing they can control most things; this belief is uncategorically proven wrong when a serious illness is diagnosed.

 Loss of control, lack of certainty, constant change: many people describe illness as an emotional roller-coaster ride. People respond to these challenges in a variety of ways. Some people try to hold on tighter; they use the skills of problem-solving that have served them well in other domains. They search the internet for all the available information on their condition and potential treatments. They consult the best medical specialists and seek second opinions. While these actions can be beneficial, they do not address the underlying issues of loss causing feelings of anxiety, fear, worry, and sadness. No matter the prognosis, there is inevitably a sense of loss, often accompanied by anger, when one is diagnosed with a physical medical condition. There may also be feelings of shame, guilt, and self-blame, especially if the condition is one directly associated with lifestyle or specific behaviors. This is without even considering the actual physical symptoms people may be dealing with: pain, sleeplessness, weakness, discomfort, loss of functional abilities, and ability to carry out activities of daily living. These symptoms tend to be more disease-specific than the common psychological sequelae of physical illness, but no less debilitating.

## 3. How Can Mindfulness Help?

 The question arises, then, of how mindfulness training can address this plethora of thorny psychosocial as well as physical issues. The beauty of a mindfulness approach is that it is eminently adaptable to a wide array of circumstances. Simply absorbing the general understanding that the only certainty in life is change, and that sometimes the best thing to do to solve a problem is nothing, can be extremely relieving and even liberating to people who are desperately and often frantically trying to fix things. Realizing that in fact they can slow down and see things as they are, without blinders, and learn ways to hold the strong emotions and sensations that arise can be transformative. The further realization that although specific symptoms may be unpleasant, they are tolerable and are too constantly in flux can provide further liberation from suffering. Hence change occurs not only through training the mind in formal meditation practice, but via a shift in attitude and perspective that allows people to see their illness in a new light, without allowing fear to consume them and drive behavior.

 Theoretically, what we see is an improvement in emotion regulation strategies employed by people with physical illness who undergo mindfulness training. They ruminate on the past and worry about the future less, and engage in less experiential avoidance of difficult feelings and situations [120]. This is possible through learning and applying mindfulness skills, particularly present-moment, nonjudgmental awareness. The downstream effects of these changes in the way people engage with their inner experience are decreases in specific disease symptomatology as well as improvements in a broad range of psychological outcomes. Unfortunately, few studies within this area look at the nuance of what may be happening to provide benefit, as the focus to date has primarily been on quantifying the nature and extent of benefits received. For a more process-related understanding, we turn primarily to qualitative studies, some of which will be summarized, and a few newer quantitative studies that are beginning to uncover potential mechanisms of action of mindfulness-based interventions, which are selectively woven throughout the following review of the literature across a wide range of physical conditions.

## 4. Summary of the Literature

In *The Art and Science of Mindfulness: Integrating mindfulness into psychology and the helping professions* [2], Shauna Shapiro and I included a chapter (Ch. 6, pp 75–91) summarizing the literature in this area up to 2008. Readers are directed there for a detailed review of studies on mindfulness-based interventions in physical health conditions to that date. The strategy employed here is to build upon that work by adding recent studies, and including a focus on levels of evidence and a high-level summary table across conditions. Indeed, there has been an explosion of work in the last four years (2008–2012) so only the seminal studies and most recent work are summarized in the text, but the table aims to be comprehensive. The literature is summarized by disease type, rather than by outcomes, since a broad range of outcomes have been studied, many of which are specific to the populations of interest. The bulk of common outcomes studied, however, are psychological, including stress levels, depression, mood states, anxiety, and other psychological reactions to illness. Others studies assessed quality of life (QL), functional abilities, and common symptoms such as pain, fatigue, and sleep. A minority of the studies looked at the impact of MBIs directly on disease pathology or progression. The bulk of the research examined the effects of MBSR interventions, so the paper will be organized by disease type rather than by type of intervention, and specific interventions detailed throughout as necessary.

The body of evidence within each condition is evaluated using a levels of evidence framework for quantitative research on intervention studies (see Table 1; [121]). In summary, the highest level of evidence (Level 1) is at least one systematic review or meta-analysis of RCTs, down to case series or pretest posttest studies without controls (Level 4). This framework will be used to summarize the quality and amount of quantitative evidence within each condition, as presented in Summary Table 2. Qualitative studies that fall outside of this framework will also be included in the text to add meat to the bones of the quantitative results.

## 5. Clinical Populations

### 5.1. Cancer

There is now a large body of work investigating the efficacy of MBIs for patients with various types of cancer (Table 2). In fact, this literature itself has been reviewed on several occasions since 2005 [122–131]. A 2009 meta-analysis of 10 studies found a medium-sized effect on psychosocial outcome variables (*d* = 0.48), but only a small effect on the handful of physical biomarker variables measured up to that point (*d* = 0.18) [128]. A more recent meta-analysis of 19 studies reported similar effect sizes on mood (*d* = 0.42) and distress (*d* = 0.48) but did not look at biological outcomes [130]. With this amount of scrutiny, level 1 evidence has accumulated on the efficacy of MBSR in cancer, specifically for improving psychological, functional, and QL outcomes, particularly in breast cancer patients [131].

#### 5.1.1. Psychological Outcomes

Because of the volume of the literature in this area, only the seminal studies and most recent large and innovative trials will be discussed. The first study in this area was published by Speca and colleagues in Canada: an RCT in which 89 patients with a variety of cancer diagnoses were randomized to MBSR or a waitlist control condition [5]. Patients in the MBSR program improved significantly more on mood states and symptoms of stress than those in the control condition, with large improvements of approximately 65% on mood and 35% on stress symptoms. They specifically reported less tension, depression, anger, concentration problems, and more vigor, as well as fewer peripheral manifestations of stress (e.g., tingling in hands and feet), cardiopulmonary symptoms of arousal (e.g., racing heart, hyperventilation), central neurological symptoms (e.g., dizziness, faintness), gastrointestinal symptoms (e.g., upset stomach, diarrhea), habitual stress behavioral patterns (e.g., smoking, grinding teeth, overeating, insomnia), anxiety/fear, and emotional instability compared to those still waiting for the program. These patients as well as the control group after treatment completion were followed-up six months later and similar benefits were maintained in both groups over the follow-up period [6]. In the combined group more home practice was associated with greater decreases in overall mood disturbance, and the greatest improvements were seen on anxiety, depression, and irritability.

Since that time many pre-post observational studies without comparison groups and RCTs similar to Speca et al. [5], with usual care or waitlist groups have been published, citing improvements in a range of outcomes including QL domains such as emotional, social, role, and physical functioning and psychological improvements on measures including stress symptoms, anxiety, depression, fear and avoidance [7–16]. Other outcomes assessed include cancer-related symptoms such as fatigue, pain, and sleep [17, 18], and existential outcomes including spirituality, posttraumatic growth, loss, and grief [19, 132], as well as various measures of mindfulness [12, 20–22].

In assessing RCTs in particular, while there are now a substantial number which compare MBIs to waitlist or usual care controls, some with quite large sample sizes [10, 12, 16, 20], still very few studies have included randomization to active comparison groups. One notable exception is a three-armed trial in which Henderson et al. [23] randomized 172 early-stage breast cancer patients into MBSR, a nutrition education program matched on contact time, or a usual care control condition, and included follow-up assessments post program (4 months) and one and two years later. The MBSR group improved more than the other two on a wide range of measures at the 4-month postprogram assessment, including quality of life, active behavioral and cognitive coping, avoidance and spirituality, as well as depression, hostility, anxiety, unhappiness, meaningfulness, and several measures of emotional control. These group differences eroded over time, however, as participants in the other two groups continued to improve more slowly, such that at 12 months MBSR was only superior on measures of spirituality, behavioral coping, and the sense of coherence subscale of “comprehensibility”, that is, making sense of one's predicament. At 24 months the only group differences apparent were on measures of anxiety, unhappiness, and emotional control, still favoring MBSR over usual care, but not the other active intervention. Nonetheless, these findings are striking and the hardest test to date of an MBI in the cancer context.

#### 5.1.2. Biological Outcomes

Another area of focus in oncology has been assessing the impact of MBIs on biomarkers, such as salivary cortisol and various measures of immune functioning. For example, Carlson et al. [24–26] measured immune, endocrine, and autonomic function pre-post MBSR in 59 breast and prostate cancer survivors by looking at the counts of a number of lymphocyte subsets, including T cells and NK cells, and T and NK cell function in response to an immune challenge. Although there were no significant changes in the overall number of lymphocytes or cell subsets, T cell production of IL-4 (an anti-inflammatory cytokine) increased when stimulated, while interferon gamma (IFN-*λ*) and NK cell production of IL-10 decreased, consistent with a shift in immune profile from one associated with depressive symptoms to a healthier profile. Patterns of change in cytokines over one year of followup also supported a continued reduction in proinflammatory cytokines [26].

Salivary cortisol profiles also shifted pre- to post-intervention, with fewer evening cortisol elevations found post-MBSR, and some normalization of abnormal diurnal salivary cortisol profiles [25]. Over the year of follow-up, continuing decreases in overall cortisol levels were seen, mostly due to further decreases in evening cortisol levels [26], which would tend to support better sleep-wake patterns. This is significant as higher cortisol levels, particularly in the evening, are considered to be an indicator of dysregulated cortisol secretion patters and poorer clinical outcomes, while flatter cortisol slopes in women with metastatic breast cancer were associated with shorter survival times [133].

Witek-Janusek and colleagues [27] investigated 38 women with breast cancer who participated in an MBSR program compared to 28 nonrandomized controls who chose not to participate, and a noncancer normative group. Both cancer groups showed altered NK cell and cytokine profiles following surgery, but the MBSR group evidenced reestablishment of NK cell activity and cytokine production toward normal levels postprogram, whereas the control group still had depleted NK cell function and activation of proinflammatory cytokines. The MBSR group also reported improvements in coping and QL relative to controls, and decreased mean late-afternoon cortisol. Lengacher and colleagues also looked at the effects of a 6-week MBSR program on lymphocyte recovery, finding increased response of T cells to antigen stimulation and an improved ratio of Th1/Th2 cytokines in early stage breast cancer [28]. They also looked at salivary cortisol and IL-6 pre-post individual MBSR classes in patients with advanced cancer and their caregivers and found decreases in both cortisol and IL-6 pre-to-post session, and also decreases in baseline levels across sessions [29]. The interpretation of these results is complex, but in general an anti-inflammatory environment is thought to be more favorable in terms of cancer outcomes than one with elevated Th2 (proinflammatory) cytokines [134].

Measures of autonomic system function have also been of interest, since cancer survivors are at high risk for cardiovascular disease due to the toxicity of their cancer treatments. Hence, Carlson et al. looked at the effects of MBSR on resting blood pressure and heart rate. In a group of breast and prostate cancer survivors, overall resting systolic blood pressure (SBP) decreased significantly from pre- to post-MBSR [26], while in another group of 72 women with various forms of cancer, weekly home BP monitoring showed significant decreases over the course of the program for women with higher premorbid BP in MBSR compared to those in a waitlist group [30]. This is desirable as high blood pressure (hypertension) is the most significant risk factor for developing cardiovascular disease.

#### 5.1.3. Modified Programs

In addition to standard MBSR and MBCT groups, other modifications of MBIs have been reported for cancer patients. These include individualized MBSR for bone marrow transplant inpatients [31, 32] and audio recordings of mindfulness practices for patients undergoing chemotherapy to use for home study [14]. A 12-session group “mindful movement” program for older women with breast cancer, based on combining dance therapy with mindfulness practice, was evaluated positively in focus groups [33], and an MBSR group intervention targeting men with advanced prostate cancer is one of the only published with an entirely male sample [34]. Another adaptation for prostate cancer is a dietary intervention supported with mindfulness practice [35, 135]. Pilot work supported the efficacy of this program for decreasing prostate specific antigen (PSA) levels in men with advanced cancer [35], while a larger RCT comparing the intervention to waitlist found improved dietary intake of vegetable proteins and decreases in fat and animal protein intake, accompanied by a slower PSA doubling time at a 3-month followup in intervention compared to control [135].

Also very innovative is a study that evaluated the effects of a brief MBCT intervention (3 classes) on sexual response, interest, and arousal in 31 women recovering from gynecological cancer [36]. Women were randomized to immediate or waitlist groups, and assessed not only by self-report, but also in a laboratory arousal paradigm. Those who participated in MBCT showed greater improvements across outcomes, although their actual objective sexual response was not different. These modified programs support the idea that mindfulness skills can be taught and applied in different formats for cancer patients and continue to provide benefit for specific subpopulations and across outcomes.

#### 5.1.4. Qualitative Studies

Qualitative research is valuable in that it helps to better understand the lived experience of participants in MBIs, fleshing out the details behind, for example, improved scores on a measure of mood or quality of life. Through interviews and focus groups, participants can describe deeply and in rich language the process of engagement and how they perceived change and benefit from learning these practices. These studies with cancer patients are described in some detail because I believe they relate to patients' experience with a wide range of physical diseases, and hence have a potentially broader application.

Several early qualitative studies set the groundwork for this line of inquiry. In the first study, Mackenzie et al. [37] interviewed nine cancer patients who had participated in the 8-week MBSR program, and who continued to attend weekly drop-in MBSR sessions, for between 1 and 6 years. With individual semistructured interviews and a focus group, five major themes were identified, labeled as: (1) opening to change; (2) self-regulation; (3) shared experience; (4) personal growth; and (5) spirituality. The interviews were used to develop specific theory concerning mechanisms whereby MBSR effects change for cancer patients. In this theory, initial participation in the 8-week program was seen as only the beginning of an ongoing process of self-discovery; a slight shift in orientation which begins the growth process. Many described it as just the “tip of the iceberg.” At the time of diagnosis patients felt isolated, scared, and unsure of what to do. The MBSR program helped to meet their needs for understanding they are not alone in their journey, taught concrete tools for self-regulation, and introduced ways to look at the world they may not have previously considered. This can result in benefits such as reduced stress symptoms and lower levels of mood disturbance. As practice progressed in the drop-in group, social support deepened as relationships further developed, and people began learning to be less reactive and exercise more diffuse self-regulation across a wider variety of life circumstances. Underlying this process is a theme of personal transformation, of feeling part of a larger whole. With this came the development of positive qualities of personal growth and positive health, often beyond merely the symptom reduction documented over the course of the initial program. A growing spirituality of finding meaning and purpose in one's life and feeling increasingly interconnected with others was part of this personal transformation. Qualities of gratitude, compassion, and equanimity may be the ultimate culmination of practice-very similar, in fact, to the goals of many of the Eastern practices upon which MBSR is based.

Similarly, Dobkin interviewed 13 women who had completed breast cancer treatment, with a focus on exploring process variables related to change over the course of MBSR [38]. The women reported taking better care of themselves, and viewing life as more meaningful and manageable. Themes identified in focus groups were: (1) acceptance; (2) regaining and maintaining mindful control; (3) taking responsibility for what could change; (4) cultivating a spirit of openness and connectedness. In identifying the processes at work for these women, alterations in levels of mindfulness and world view were highlighted. In another sample of women with early stage breast cancer, Weitz et al. [39] conducted a phenomenological analysis of 8 women who had participated in MBSR. Themes identified included: (1) the cancer journey: a shift in perception; (2) the treatment journey: the experience of MBSR; (3) the journey toward recovery; (4) the journey toward self. The first theme described the effects of a cancer diagnosis and treatment in general, while MBSR effects were described in the second theme. These include the importance of attitudes of acceptance, letting go, and nonjudging in helping women cope with the uncertainties and fears of cancer. In the journey towards recovery a theme of “try anything” led to their exploration of mindfulness and other complementary approaches, and the journey towards self highlighted the ideas of spirituality, letting go, and lessening the identification of self as a cancer patient, rather moving towards one's own authentic self.

More recent international qualitative work also highlighted similar themes. Ando et al. [40] interviewed 28 Japanese participants after just two mindfulness-meditation sessions and identified some similar themes to Mackenzie et al. [37]. However, they highlighted that the concepts around spirituality and self-control were not as predominant in their Japanese group compared to previous Western samples. The focus was on adaptive coping, personal growth, and finding positive meaning in their cancer experience. Kvillemo and Branstrom [18] reported on 18 Swedish patients who participated in an 8-week MBSR program, and interestingly interviewees expressed not only positive outcomes, but several also struggled with particular practices, and some expressed that they received little benefit. This highlights that the program is not for everyone, though it is rare to find this discussed in most papers to date. Overall, participants described feeling increasingly calm, having more energy, less physical pain, improved sleep, and increased well-being after the program. Components emphasized as helpful were the nonjudgmental and accepting approach of the program and instructors, the influence of the group process and sharing experiences with others who have had similar experiences, as well as the emphasis on increased awareness of the present moment.

In summary, the literature in cancer and MBIs is substantial and continues to grow, with improving quality of research design through the application of active control groups, larger samples, more diverse patient groups, longer followup, and a wide range of outcomes. Strong level 1 evidence has accumulated supporting the efficacy of a range of MBIs for improving psychological well-being and overall quality of life. Qualitative research has helped to explicate the processes at work for participants and tie the quantitative results to the theory of mindfulness and its application to people living with cancer.

### 5.2. Pain

The earliest reported application of MBSR was for patients with chronic pain [49], and work has continued to grow in this area. Other more specific pain conditions have also been studied, including low back pain, fibromyalgia, rheumatoid arthritis, and migraine. Recently this literature has been reviewed in depth on several occasions [136–140]. Chiesa and Serretti [137] systematically reviewed all controlled studies (*n* = 10) and concluded that MBIs could have nonspecific effects on mood, coping, and pain symptoms in chronic pain patients, but that the studies often suffered from small sample sizes, lack of randomization and the use of nonspecific control groups. Veehof et al. [139] conducted a meta-analysis of both controlled and uncontrolled studies (*n* = 22) and found effect sizes of 0.37 for pain improvements and 0.32 for depression (small effects). Veehof suggested that while MBSR and ACT interventions for chronic pain can be good alternatives, they are not superior to standard CBT for pain [139]. In all, the level of evidence in this area is rated at strong level 2, as there are a few well-designed RCTs with positive outcomes, but results are sometimes inconsistent and there is not a conclusive positive meta-analysis of RCTs to warrant a Level I efficacy designation.

A number of cross-sectional or process studies in chronic pain have also looked at associations between mindfulness and acceptance constructs, psychological flexibility, catastrophizing, self-compassion, and pain symptoms [50–55, 141–143], but since these studies did not primarily evaluate interventions, they will not be reviewed in detail. In addition, a number of laboratory and neuroimaging studies have investigated pain and mindfulness in healthy participants and meditation practitioners [144–153], which will also not be a focus. This section covers clinical intervention studies of the efficacy of MBIs for pain control and is organized by the type of pain condition.

#### 5.2.1. Chronic Pain

 The earliest reports from Kabat-Zinn and colleagues focused on patients with chronic pain syndromes [49, 56, 57]. The 1982 report evaluated 51 patients before and after MBSR participation, documenting improvements in pain levels as well as mood and other psychiatric symptoms. A similar pre-post design was used in a larger sample of 90 patients (including the original 51) and showed similar improvements pre- to post-intervention. Kabat-Zinn and colleagues also compared these MBSR participants to a nonrandomized treatment as usual (TAU) group from the pain clinic, finding that the MBSR patients had improved more than the TAU group on measures of mood, pain symptoms and general psychiatric distress. They then conducted a series of follow-up assessments in 1987 with 225 chronic pain patients who had completed MBSR over a several year period, showing that although pain ratings themselves had returned to baseline within about 6 months, other ratings of general distress, psychological symptoms and adherence to the mindfulness practices was maintained. Other pre-post studies showed similar improvements on measures of ratings and beliefs about pain, as well as improved mood, fewer psychiatric symptoms [58] and decreased pain intensity, functional interference, improved QL and distress [59]. Participants in a “Breathworks” mindfulness-based program also showed improvements on measures of acceptance, depression, outlook, catastrophizing, and pain self-efficacy compared to pain patients who chose not to participate in the group [60].

 McCracken et al. studied the efficacy of ACT for chronic pain in several uncontrolled studies, first reporting on a group of 108 chronic pain patients who participated in a 3-4 week residential pain program [61]. Overall participants reported increases in emotional, social, and physical functioning, and decreases in healthcare utilization. This team then replicated their findings in a new sample of 171 chronic pain sufferers, offering three to four weeks of ACT and mindfulness-based treatment in a group format, 5 days per week for 6.5 hrs each day. Significant improvements were reported pre- to posttreatment on measures of pain, depression, pain-related anxiety, disability, medical visits, work status, and physical performance [62]. In a shorter out-patient ACT program, two small pilot samples showed improvements in acceptance, pain, disability, depression, and anxiety [154].

 Only two studies have employed randomized comparison groups. Plews-Ogan et al. [63] evaluated MBSR and massage for the management of chronic musculoskeletal pain in 30 pain patients randomly assigned to either MBSR, massage or a no-intervention control condition. Immediately postintervention, the massage group had more pain reduction and improved mental health status compared to usual care, while the MBSR group showed greater longer-term (1 month) improvements in mental health outcomes compared to usual care and the massage condition [63]. Thus, MBSR was more effective for enhancing mood in the long term, but massage provided more immediate pain relief.

Finally, Wong et al. [64] conducted the largest trial to date, comparing two active interventions for 99 chronic pain patients in Hong Kong, MBSR and a multidisciplinary pain intervention, composed of primarily psychoeducation with sessions on physiotherapy for pain and nutrition. In both groups, patients who completed the interventions improved similarly on measures of pain intensity and pain-related distress over time. Without a usual care or no-treatment control, it is difficult to conclude if these improvements are due to the interventions themselves or simple fluctuations in symptomatology due to natural healing over time, historical trends, regression towards the mean or expectancy effects. Hence, the cumulative evidence in this area remains a weak level 2 for efficacy in the management of general chronic pain.

#### 5.2.2. Low Back Pain

 More specifically, MBSR has been successfully applied to the treatment of chronic lower back pain. Carson et al. applied a variant of MBSR with extended focus on loving-kindness practice with 43 patients [67]. They were randomly assigned to the loving kindness program or TAU; treatment participants showed improvements in pain perception and psychological distress, whereas there were no improvements in the control group. In older adults, Morone et al. [68] recruited 37 participants with an average age of 75 years who were randomized to MBSR or a waitlist control condition. Compared to the waitlist, the MBSR participants showed significantly greater improvements on measures of chronic pain acceptance, engagement in activities, and overall physical functioning. Another application to chronic low back pain utilized an intervention called “breath therapy,” which combines body awareness, breathing, meditation, and movement, similar to the MBSR program [69]. Thirty-six patients with chronic low back pain were randomized to breath therapy or standard physical therapy, and assessed at baseline, postassessment and 6 months later. Both groups of patients reported improved pain, and those in breath therapy had greater improvements in functional, physical, and emotional role performance, while those in physical therapy improved more in vitality.

 A qualitative study by Hsu et al. [70] interviewed 18 chronic low back pain patients participating in MBSR and identified several areas of change, including improved emotional states, changes in ways of thinking about their pain, more relaxation, greater levels of mindfulness, increasing their options for healing, and inciting feelings of hope for the future.

 Although sample sizes in the RCTs conducted in this area remain small, the support for MBSR as a helpful intervention for improving coping with pain symptoms and overall adjustment in chronic low back pain patients continues to mount, with solid level 2 evidence currently available. One review paper from 2009 supports this conclusion [140].

#### 5.2.3. Fibromyalgia

 Fibromyalgia (FM) is another pain-related condition associated with overall bodily stiffness, soreness, and pain trigger points located throughout the body, where symptoms seem to be exacerbated by stress. Other symptoms include fatigue and sleep disturbance, and fibromyalgia is considered notoriously difficult to treat. An early report in 1993 with pre-post assessments of 59 participants in MBSR showed improvements on scales of well-being, pain, fatigue, sleep, coping and FM symptoms, as well as general psychiatric symptomatology [71]. Patients were classified as “responders” if they showed moderate to marked overall improvement. By this definition, 51% of the sample responded to the treatment. Other nonrandomized studies provide further support. A comparative study assessed MBSR compared to both a waitlist group and those not interested in the group at all [72]. There were greater improvements in the MBSR group on measures of pain, sleep, FM impact and global severity of psychological symptoms. Similarly, Grossman et al. [74] used a quasi-experimental design assigning 58 women to either MBSR or an active support condition based on date of entry into the study. The women in the MBSR condition showed greater improvement pre-post on measures of pain, coping, quality of life, anxiety, depression and somatic complaints, which were maintained 3 years later.

RCTs have also been conducted in this condition assigning patients to either MBSR or waitlist control groups. Weissbecker et al. [75] reported an increased sense of coherence (the disposition to experience life as meaningful and manageable) in 91 women with FM assigned either MBSR (*n* = 51) or waitlist control (*n* = 40) groups, which was related to lower levels of perceived stress and less depression. Sephton et al. [155], reporting on the same study, assessed depression pre-post MBSR and two months later. Those in the MBSR group showed significantly more improvement on depression symptoms across all three time points. A comparison of a Yoga of Awareness program (which included meditation, breathing exercises, group discussion, as well as yoga postures) to usual care reported greater improvements in both pain levels and mood, as well as a range of coping strategies in the intervention group [76].

Other researchers have also compared MBIs to other active control conditions in fibromyalgia, a more rigorous test of specificity. Astin et al. [77] randomly assigned 128 patients with FM to a group combining mindfulness meditation plus Qigong movement therapy or an education support group control condition. A large number of outcome measures including pain, disability, depression, and coping were assessed, but although patients did improve over time, neither group proved superior. In the most rigorous study to date [78], 177 female patients were randomized to: (1) MBSR, (2) an active control condition, or (3) wait list. The active control was matched to MBSR on format, instructors, contact time and homework, with the focus on progressive muscle relaxation and stretching, rather than meditation and yoga *per se*. There were no significant differences between groups on the primary outcome of health-related quality of life two months posttreatment, but patients improved across conditions over time. Post hoc analyses showed that only MBSR resulted in a significant pre-to-post-intervention improvement in quality of life, and on 6 of 8 secondary outcome variables compared to improvements on three measures for the active control group, and two in the wait list condition.

 Hence, MBSR seems to be an effective intervention for alleviating symptoms common in FM such as pain, depression, and a range of psychological outcomes, although it has not proven superior to other active control conditions, and not yet been tested against proven efficacious treatments such as cognitive behavior therapy, that would provide a tougher test of overall efficacy. The level of evidence at this stage is rated at weak Level 2.

#### 5.2.4. Rheumatoid Arthritis

There are two RCTs in this area investigating the effect of MBSR on RA, a painful autoimmune condition caused by swelling of the joints [79, 80]. In the first, 63 participants were randomized to MBSR or a waitlist control condition. After two months, there were no differences between the groups on measures of depressive symptoms, psychological distress, well-being, mindfulness, and RA disease activity as evaluated by a physician masked to treatment status. However, at 6 months there were significant improvements across self-reported outcomes in the MBSR group.

The second study employed multimodal outcome measures and compared 144 participants randomly assigned to one of three conditions: cognitive behavioral therapy (CBT) for pain; mindfulness meditation and emotion regulation therapy; education-only, which served as an attention placebo control [80]. The greatest improvements in pain control and reductions in inflammatory cytokines were observed in participants in the CBT pain group, but both the CBT and mindfulness groups improved more in coping efficacy than the education control group. Most strikingly, patients with a positive history of depression benefited more from mindfulness on outcomes of both negative and positive affect and physicians' ratings of joint tenderness, suggesting that MBSR might be preferable to CBT for treating individuals who struggle with depression.

 Finally, a qualitative study reported on focus groups with MBSR participants and used phenomenological analysis to derive two major themes: responding to pain and psychological well-being [81]. Participants described a changing relationship to pain, stating that it no longer dominated their life, overwhelmed them, and restricted their activities. Instead, more responsive approaches to pain were described, including engaging with pain, rather than trying to resist or deny its existence. This resulted in improved psychological well-being, particularly less depression.

In summary, the state of the evidence for RA is rated at weak level 2, due to inconsistent findings on primary outcomes reported in large relatively well-designed RCTs. Only with secondary analyses did outcomes emerge consistently favoring MBSR.

### 5.3. Cardiovascular Disorders

 A good deal of meditation research using the transcendental mediation (TM) approach has looked at patients with hypertension (high blood pressure) and coronary heart disease (CHD) and reported positive findings (e.g., [156–158]). This research has been reviewed elsewhere [159, 160] and here we will focus only on recent applications of MBIs. In addition, a range of studies have looked at the effects of MBIs on variables potentially important for the development of cardiovascular disorders in largely healthy samples, such as heart rate control, blood pressure, heart rate variability, and laboratory stress reactivity, [161–165], which are not included in this review. Studies have also looked at the effects of MBIs for treating people with clinically elevated blood pressure, which will be included.

In addition to pre-post studies showing improvements on a range of psychological outcomes in people with heart disease [82–84], two very small RCTs comparing MBSR to waitlist control groups were reported by Tacón and colleagues [85, 86], who reported benefits in anxiety, emotional regulation and less use of reactive coping styles in MBSR participants, as well as slower breathing frequency post-interventions in a laboratory stress test. In another study, 19 very ill elderly patients with congestive heart failure were randomized to a meditation group which participated in weekly sessions and listened to a meditation tape at home for 30 minutes, twice daily, for 12 weeks, or a control group that only attended weekly meetings [87]. The meditation group showed significantly greater improvements on measures of quality of life, lower levels of norepinephrine, and better cardiopulmonary performance on exercise testing.

Sullivan and colleagues conducted a novel prospective cohort study (SEARCH) in which they geographically assigned 208 patients with chronic heart failure to either a MBI consisting of mindfulness meditation practice, coping skills and group discussion for those who lived close enough to attend, or a usual care control condition for those living further away from the medical centre [88]. Patients in the MBI had greater decreases in anxiety, depression, and cardiac symptoms postintervention and at 3 and 6 months. After one year of follow-up group differences were no longer apparent, however, as all participants had increased symptoms.

A qualitative study of 6 participants in an MBCT cardiac rehab group [89] identified five central themes: development of awareness, commitment, experiences within the group, relating to the material, and acceptance. These included sub-themes such as awareness of thoughts, of cardiac symptoms, and of mind-body interactions. Group factors such as normalizing one's experience, a sense of shared group identity, and the empathic and anxiety relieving nature of the group setting were considered helpful. As in previous studies, not all participants connected with all the techniques and approaches, so the importance of finding those that did seem to mesh with each person was emphasized, as well as the importance of adopting a committed attitude and finding time to fit the practices into busy lifestyles. Most also commented on improved mood as an outcome.

Studies have also evaluated the impact of MBIs on blood pressure in hypertensive or prehypertensive patients. A 2012 review of 14 studies (13 of which were RCTs) concluded small yet meaningful reductions in both systolic and diastolic BP could be reliably achieved through meditation interventions [166]. For example, a well-designed study of 52 patients with hypertension randomized participants to contemplative meditation practice or a no-treatment control and found decreases in heart rate, and both systolic and diastolic blood pressure as measured during 24-hour ambulatory monitoring and in reaction to mental stress testing [90]. As well, a recent study comparing the effects of MBSR to progressive muscle relaxation in 56 prehypertensive adults found that MBSR produced significant reductions in SBP and DBP compared to the relaxation control group [91].

Currently, level 1 evidence exists for the value of MBIs in reducing blood pressure in people with cardiovascular disorders, and level 2 evidence for improving a broad range of psychological outcomes including anxiety and depression, quality of life and coping.

### 5.4. Diabetes

Quite a few studies have been reported in this area recently, many of which have monitored biomarkers as well as psychological functioning. A pilot study in 11 patients with type II diabetes investigated the impact of MBSR on indices of glycemic control, given that stress has been related to poorer control of blood sugar levels in this group [93]. At one month following the program, glycosylated hemoglobin A1C (HbA1C), the measure of blood sugar levels, was significantly reduced, as were measures of blood pressure, depression, anxiety, and overall psychological distress.

In addition to one pre-post study of 25 patients which showed improvements across mood scales [94], several studies have compared MBIs to randomized control conditions. A small RCT compared MBSR to nutrition control in 20 patients [95] and investigated effects on neuropathic pain, QL, and sleep. Quality of life related to pain and symptoms improved more in the MBSR group, but these findings were considered quite preliminary. In a larger RCT, Gregg et al. [96] randomized 81 patients to either ACT plus a one-day educational workshop or to education only for managing type II diabetes. Those who participated in ACT therapy were more likely to use adaptive coping and report better self-care behaviors. They were also more likely to have glycosylated hemoglobin A1c values in the target range. In the largest study to date [97], 110 patients with type II diabetes were assigned to either MBSR or a TAU control, and will be followed for 5 years on both psychological and biological outcome markers. At the first year of followup, MBSR participants improved more on depression and health status overall, and in stress symptoms for those who completed the treatment. One other RCT comparing MBSR to waitlist control has been reported only as a protocol at this time [98].

In summary, there is level 2 evidence that MBIs can be beneficial for improving psychological functioning and possibly improving glycemic control and helping with the neuropathy associated with type II diabetes.

### 5.5. Human Immunodeficiency Virus (HIV)/Acquired Immune Deficiency Syndrome (AIDS)

Research in this area has increased vastly in the last four years. In terms of preliminary work, a pilot study of MBSR in HIV-infected youth sought to determine the feasibility of this type of intervention in a small group of 13–21 year olds [99]. Interviews with those who completed the program identified five themes: (1) improved attitudes (less negativity); (2) decreased reactivity and impulsivity; (3) improved behavior, less lashing out; (4) improved self-care; (5) the value of being in a group. On average, they rated the importance of the group to them at 9.6 on a scale from 1 to 10, indicating that although attrition was high, those HIV-infected youth who were able to complete the program did benefit.

In terms of RCTs, an innovative study with 58 late-stage AIDS patients in a palliative care setting randomized participants to one month of loving-kindness (Metta) meditation, massage, both, or neither [100]. The meditation was self-administered through an audiotape. The combined meditation and massage group showed the most benefit in terms of greater overall quality of life and spirituality compared to either treatment alone. Gayner et al. [101] randomized 117 men with HIV to either MBSR or TAU. Compared to the TAU group, MBSR participants had significantly lower scores of avoidance and higher positive affect postprogram. They also had higher scores on the Toronto Mindfulness Scale on curiosity and decentering, both postprogram and at a 6 month followup.

Another RCT sought to assess the potential for MBSR to help patients cope with common side-effects of antiretroviral medication, including gastrointestinal problems such as diarrhea, nausea, and vomiting, neuropathic pain and dermatological problems such as rashes [102]. Seventy-six people living with HIV were assigned to MBSR or waitlist control, and assessed post-program and over a 6-month follow-up period. The MBSR group had fewer symptoms related to antiretroviral therapies at both post-program and follow-up, as well as less symptom-related distress.

Three studies have investigated the impact of MBSR on immune measures in HIV positive participants. A small pilot study of HIV positive men in Iran showed improvements in psychological outcomes post-MBSR and over 12 months of follow-up, as well as increases in CD4 T-cell lymphocyte numbers [103], a primary marker of disease progression in HIV. However, there was no comparison group. In another study using a nonrandomized design, 46 HIV-infected patients were recruited for either the MBSR or comparison groups (assigned by patient preference), but only 24 completed the study [104]. Participants in the MBSR group compared to the controls showed an increase in NK cell activity and number, an important measure in HIV infection, since NK cells represent the main type of innate immunity in the body and help to fight off opportunistic viral infections.

In an RCT, Cresswell and colleagues [105] assigned 48 HIV infected adults to either an 8 week MBSR class or a 1-day stress reduction education seminar control condition. Peripheral counts of CD4+ T lymphocytes decreased substantially in the control group, but remained stable in the MBSR group. Class attendance mediated the effects of MBSR on buffering T-cell decreases over time, such that those who attended more classes showed more stability in their counts, accounting for up to 2/3 of the effect on T-cell counts.

In sum, there is level 2 evidence from RCTs that MBSR in HIV-infected men helps to improve psychological well-being as well as improve measures of immune system functioning that are important predictors of disease progression.

### 5.6. Irritable Bowel Syndrome

Prior to 2010 there were no reports in the literature on the application of MBIs for treating symptoms and well-being for people with irritable bowel syndrome (IBS), but since that time several research groups have reported large, well-designed, and controlled RCTs. IBS is arguably a good target for what MBIs have to offer, as it is a functional psychosomatic disorder that is exacerbated by stress and has no discernible physiological cause. The first treatment results were published by Ljótsson and colleagues in Sweden, who have conducted much of the work in this area. The intervention they evaluated is a 10-session group focusing on three themes: cognitive awareness and education around stress and coping, mindfulness training and exposure to noxious IBS symptoms. In a pre-post pilot study of 34 patients they reported improvements in IBS symptoms, quality of life, anxiety, and overall functioning [106]. Another pre-post study of traditional MBSR reported similar gains in 93 patients on IBS-specific quality of life and visceral anxiety [107].

 Since that time several RCTs have compared MBIs to a variety of mostly active control groups. The Swedish group continued by conducting an RCT of an online adapted version of their acceptance and mindfulness-based intervention with 61 patients, comparing it to waitlist, and assessing psychological, physical, and health economic outcomes [108]. The intervention was conducted largely by patients on their own at home, with weekly internet contact with therapists via e-mail. There was also a closed discussion forum for patients to share questions or progress with one another. Participants had to work through 5 successive therapeutic steps and were not able to move to the next step until having completed the previous one. Compared to those on the waitlist, participants improved more over time on IBS symptom severity, quality of life, and IBS-related fear and avoidance behaviors, which were maintained over 12 months. In addition, the intervention was more cost-effective than the waiting list, with an 87% chance of leading to reduced societal costs.

 This group continued investigating the online MBI against active control groups, randomly assigning 86 patients to either the online acceptance and mindfulness intervention or an online discussion forum waitlist [167]. In this case, participants in the treatment condition reported a 42% decrease (compared to a 12% increase) in primary IBS-symptoms, and they improved on GI-specific anxiety, depression, and general functioning with large effect sizes. These participants were followed up 16 months later after they had all completed the intervention [109]. Treatment gains were maintained on all outcome measures, including IBS symptoms, quality of life, and anxiety related to gastrointestinal symptoms, again with large effect sizes (most *d* > 1.0). About 60% of the total sample reported clinical relief of symptoms. Another followup on this sample reported cost reductions of $16,806 per successfully treated case, driven by reduced work loss in the treatment group [110].

 Finally, this group investigated their online mindfulness intervention compared to online stress management matched in time and format in a large RCT with 195 patients, and also measured credibility of the treatments, expectancy for improvement and therapeutic alliance [111]. At posttreatment and 6-month follow-up, the MBI group improved more than stress management on IBS symptom severity, IBS quality of life, visceral sensitivity, and the cognitive scale for functional bowel disorders. Both groups improved similarly on the perceived stress scale and hospital anxiety and depression scale subscales. There were also no group differences on the treatment credibility scale or the working alliance inventory. This impressive series of studies provides strong support for the efficacy of the online MBI for improving both physical symptoms and QL in IBS patients.

 Another group from the USA has tested in-person traditional MBSR in 75 female IBS patients, reporting an RCT comparing MBSR to a support group matched for time and other nonspecific factors [112]. Women in MBSR, compared to the support group, showed greater reductions in IBS symptom severity posttraining (26.4% versus 6.2% reduction) and at 3-month follow-up (38.2% versus 11.8%). Changes in quality of life, psychological distress, and visceral anxiety favoring MBSR emerged only at the 3-month follow-up. Path analysis suggested that MBSR worked by promoting nonreactivity to gut-focused anxiety and less catastrophic appraisals of the significance of abdominal sensations, as well as refocusing attention onto interoceptive data without the high levels of emotional reactivity often characteristic of the disorder [113]. Essentially, these are the predicted results of exposure coupled with acceptance, as also described by the Ljotsson group.

 In the most recent study, an RCT of MBSR versus waitlist for 90 IBS suffers, Zernicke et al. [114] showed that while both groups exhibited a decrease in IBS symptom severity scores over time, the improvement in the MBSR group was greater than the controls and was clinically meaningful, with symptom severity decreasing from constantly to occasionally present. This clinically meaningful improvement in symptoms was maintained in the MBSR group 6 months later.

Considered together, this body of well-designed and executed studies provides consistent level 2 evidence for the efficacy of both in-person and online versions of MBIs for IBS sufferers. Once studied together in a review or meta-analysis of RCTs level 1 evidence could be established.

### 5.7. Organ Transplant

A series of well-designed studies has been conducted by Gross and colleagues for patients who were recipients of organ transplants [116–118]. Pilot work with 19 kidney, lung, or pancreas transplant recipients showed improvement from baseline after MBSR on measures of depression and sleep, with the sleep effects maintained at 3-month followup, when improvements in anxiety also became significant [116]. At 6-month post-MBSR, continued improvements in sleep quality and duration as well as anxiety and depression were reported [117]. In a larger RCT, 138 recipients of kidney, kidney/pancreas, liver, heart, or lung transplants were randomized to either MBSR or a health education control group. MBSR participants had greater reductions in anxiety and sleep symptoms compared to the controls, with medium-sized effects up to one-year follow-up. Within the MBSR group, anxiety, depression, and sleep symptoms decreased and quality-of-life improved by 8 weeks; these benefits were retained at 1 year. Hence, level 2 evidence suggests MBSR is an effective treatment for psychological symptoms in organ transplant recipients.

### 5.8. Other Diagnoses

A number of studies have investigated the potential efficacy of mindfulness-based interventions in a wide variety of other medical conditions. Due to space limitations these will be mentioned but not covered in detail; see Shapiro and Carlson [2] for a more detailed summary to that date. Other areas that may be classified as medical conditions are also not covered in this paper, including pregnancy [168–170], obesity [171–176], and insomnia [177–179] but it is worth mentioning that there is growing research on the application of MBIs for these conditions.

To give a sense of the breadth and range of additional applications, studies have been published evaluating MBIs for epilepsy [180–183], psoriasis [184], multiple sclerosis [185, 186], tinnitus (a constant ringing in the ears) [187, 188], King-Kopetzky syndrome (characterized by difficulty hearing speech in the presence of background noise) [189], menopause-related hot flashes [190, 191], prader-willi syndrome (characterized by over-eating and delay in the satiety response) [192], hepatitis C, [193], asthma [194], Parkinson's disease [195], Tourette's syndrome [196], chronic fatigue [197], urinary incontinence [198], failed back surgery syndrome [199], aneurismal subarachnoid hemorrhages [200], memory loss [201, 202], chronic obstructive lung disease [203], vestibular dysfunction [204], sexual dysfunction [205], migraines [206], as well as for groups of mixed medical patients [207–211].

Overall, these studies have varied in quality and outcomes, but indicate potential or demonstrated benefit for people suffering from this wide array of conditions across outcomes such as depression, anxiety, quality of life, coping, and specific symptomatic relief.

## 6. Conclusions

 This paper began with a theoretical discussion of the experience of suffering chronic or acute illness, and how MBIs might help people coping with a range of conditions. This was followed by a review of the specific conditions with the most empirical evidence to support their efficacy. As can be seen from Table 2, there are solid RCTs supporting the efficacy of MBIs across a broad range of diseases. This brings us full-circle to the idea of why MBIs are beneficial, and how they may work. There are a number of studies mentioned throughout the paper that have focused on process or mechanisms of change, looking mostly at mediating factors such as the development of mindfulness and acceptance.

Indeed, in the face of physical symptoms which are exacerbated by stress (e.g., as is the case with IBS) or avoided as paying attention to them produces anxiety (again for IBS and some pain conditions), mindful attention and exposure are likely important mechanisms of change. Learning to identify the stress response and associated symptoms, and respond mindfully rather than react with aversion, can result in acceptance and relaxation which will then result in symptom attenuation rather than increasing severity. For diagnoses which are the cause of considerable worry, anxiety and life threat (e.g., as in the case of cancer, HIV/AIDS, and cardiovascular disease) processes such as acceptance and emotion regulation strategies that result in reduction in worry and rumination are likely important.

The universality and consistency of results across conditions points to these potential shared mechanisms of action that may rely on the same underlying processes, regardless of the particular disease presentation. While there are likely nuances to this (i.e., in HIV patients T-cells may be affected; in diabetes blood sugar levels adapt; in autoimmune and pain conditions inflammation may be affected, etc.) the underlying processes of change share universalities inherent across mindfulness-based approaches.

In terms of recommendations for research directions in this area, further focus on tailoring interventions to individuals would be helpful; for example determining goodness-of-fit or treatment matching, as not everyone benefits from mindfulness-based approaches. Continued comparisons to gold-standard active interventions would constitute more difficult tests of the specificity of MBIs. As seen in some of the pain studies, MBIs may not prove superior to other cognitive-behavioral approaches, which is important to know. Nonspecific factors such as group support, the therapeutic alliance, expectancy for improvement, psychoeducation, self-monitoring, and self-empowerment are likely also important drivers of change. Further research evaluating mechanisms of change, processes of change, and mediating factors would help improve understanding of what is happening within these complex multidimensional interventions. Further adapting and tailoring MBIs to individual interventions, home-study programs, online adaptations, and investigating the efficacy of shorter groups would also be beneficial to reach larger groups of underserved patients in rural and remote locations. At the same time, treatment integrity is essential, including the training of the professionals delivering the interventions. The principles of training facilitators who are grounded in the practices, practice themselves and receive adequate training and supervision continue to be essential, and in fact are likely more important as various adaptations in form and delivery continue to evolve.

This continual evolution of MBIs and application across a broad range of physical conditions may be worrisome to some practitioners, but the root motivation of these practices is to increase compassion and decrease suffering in as many people as possible. Based on the research reviewed in this paper, these adaptations appear to be working towards just that. Therapists and researchers should be encouraged to continue this important work by applying their own mindfulness skills and deep contemplation to the design and execution of studies aimed at reducing suffering in those afflicted by difficult physical conditions.

## Figures and Tables

**Table 1:** Levels of evidence framework.

Level	Description
1	Systematic review/meta-analysis of RCTs
2	Randomized controlled trial (using usual care, waitlist, or active control group)
3.1	Pseudorandomized Controlled Trial (i.e., alternate allocation or some other method)
3.2	Comparative study with concurrent controls (e.g., nonrandomized experimental trial; Cohort study; case-control study; interrupted time series with a control group)
3.3	Comparative study without concurrent controls (e.g., historical control study; two or more single arm study; interrupted time series without a parallel control group)
4	Case series/pretest, posttest

**Table 2:** Summary of conditions.

Condition	Studies	Methods and outcomes	Comments
Cancer	[5–48]	- Early studies were pre-post evaluations of change over time; showed improvements in psychological constructs: stress symptoms, mood, quality of life, depression - Biomarker studies: decreases in proinflammatory cytokines, cortisol levels - Symptoms: improved sleep, less fatigue, less pain - Later multiple RCTs confirmed results against waitlist, usual care and active controls. Some large samples (*n* > 200) - Qualitative work describes benefits in terms of personal growth, spirituality, coping, and shifting perspectives - Several modifications on traditional group-based MBSR such as fewer sessions, individualized treatments, and home-based audiotape interventions also showed benefit - Some mechanistic work suggests improvements related to decreased rumination, worry, and experiential avoidance	- Strong level 1 evidence for the efficacy of MBSR for improving anxiety, depression, stress, QL, and general well-being in cancer patients. - Weaker evidence for effects on health outcomes and biomarkers - This area is one of the most developed in terms of physical conditions, progressing from small uncontrolled trials to large RCTs against active interventions and multiple narrative and quantitative reviews - Most research still in breast cancer need to expand into other groups

Chronic Pain	[49–66]	- Early series of pre-post studies by Kabat-Zinn and colleagues showed improved pain outcomes and mood, which were larger than a nonrandomized TAU comparison - A series of studies on ACT for chronic pain showed improvements in mindfulness and acceptance which accounted for change in pain outcomes - Only one RCT in this area found MBSR to be superior to another treatment, one other found it comparable to a standard multidisciplinary pain intervention	- Weak level 2 evidence - 1 very small positive RCT; 1 inconclusive; the bulk are pre-post - Lots of work looking at processes of change, improvements in mindfulness and associations with pain scores

Low Back Pain	[67–73]	- RCTs comparing MBIs to TAU or waitlist showed improved pain, distress, acceptance, functional, physical, and emotional role performance - Qualitative work shows improvements in hope, relaxation, mindfulness, and ways of relating to pain	- Level 2 evidence for efficacy (Multiple small RCTs) - Interventions were varied: loving-kindness, MBSR, breath therapy - Short or no follow-up periods

Fibromyalgia	[71, 72, 74–78]	- Studies show improvements in sleep, pain, fatigue, coping, in both pre-post and comparisons to nonrandomized groups - Improvement in sense of coherence compared to waitlist - Two RCTs did not find differential benefit of mindfulness versus education support or active controls both groups improved	- Weak level 2 evidence - Not clearly superior to other active control conditions

Rheumatoid arthritis	[79–81]	- 2 RCTs comparing MBIs to CBT and education controls - Primary outcomes typically showed no group differences immediately postinterventions, but on longer follow-up MBSR was superior, and for patients who were depressed at baseline	- Weak level 2 evidence - Nicely controlled and designed studies with large Ns.

Cardiovascular disease	[82–92]	- Pre-post studies and small RCTs support MBIs benefits for improving psychological function, emotion regulation, quality of life and coping - 13 RCTs support efficacy of MBIs for decreasing blood pressure	- Level 1 evidence for MBIs in reducing blood pressure - Level 2 evidence for improving psychological functioning including anxiety, depression, quality of life and coping

Diabetes	[93–98]	- Small and larger RCTs comparing MBIs to waitlist, TAU and active controls showed improved psychological function (depression, anxiety), coping, self-care activities - Also showed improvements in glycemic control	- Level 2 evidence for efficacy in reducing anxiety and depression - Weak level 2 evidence for improving indices of glycemic control and neuropathic pain.

HIV/AIDS	[99–105]	- RCTs show efficacy for MBSR improving side-effects of antiretroviral treatment, psychological well-being - One RCT and two nonrandomized studies showed improvement or stability in T-cells and NK cells	- Level 2 evidence for improving psychological functioning, coping with side-effects and stability of CD-4 T-cell counts

Irritable bowel Syndrome (IBS)	[106–115]	- Series of research on an online MBI focusing on acceptance, mindfulness, and exposure therapy has shown efficacy in multiple RCTs for improving IBS symptoms and quality of life, as well as cost-savings, over 1-year of followup - Other RCTs of in-person MBSR confirm its efficacy for symptom reduction	- Strong level 2 evidence for improving IBS symptoms, anxiety and, quality of life both in person and online - Much of the research has studied online mindfulness and acceptance therapy

Organ transplant	[116–119]	- Pilot pre-post work followed by one large RCT showed improvements in anxiety and sleep compared to an education control	- Level 2 evidence, but based on only one well-designed RCT
